# Carotid intima-media thickness and left ventricular hypertrophy in hemodialysis patients

**DOI:** 10.12861/jrip.2013.42

**Published:** 2013-10-10

**Authors:** Mahmoud Rafieian-Kopaei, Hamid Nasri

**Affiliations:** ^1^Medical Plants Research Center, Shahrekord University of Medical Sciences, Shahrekord, Iran; ^2^Department of Internal Medicine, Shahrekord University of Medical Sciences, Shahrekord, Iran

**Keywords:** Hemodialysis, Intima–media thickness, End-stage renal disease

## Abstract

**Introduction:** Two principal findings of cardiovascular disease in end-stage renal disease patients undergoing regular hemodialysis are left ventricular hypertrophy (LVH) and arterial disease due to rapidly progressive atherosclerotic vascular disease that can be characterized by an enlargement and hypertrophy of arteries (intima-media complex thickening; IMT).Objectives: In this study, we sought to evaluate the relationship between left ventricular hypertrophy with intima-media complex thickening in end-stage renal disease patients undergoing regular hemodialysis.

**Patients and Methods:** Sixty-one patients with end-stage renal disease (ESRD) who were undergoing regular and maintenance hemodialysis treatment (F=23, M=38) were studied. The subjects consisted of 50 non-diabetic hemodialysis patients (F=20, M=30) and 11 diabetic hemodialysis patients (F=3, M=8). For all the subjects, echocardiography and carotid intima-media thickness measuring by B-mode ultrasonography were performed.

** Results:** In this study, there was a positive correlation between stages of LVH with duration of hemodialysis treatment, stages of hypertension (HTN), and with carotid-IMT. A positive correlation was also seen between stages of LVH and presence of chest pain, and more thickening of the intima-media complex was seen in the diabetic group. Diabetes mellitus was associated with the presence of chest pain, as was positive correlation between stages of HTN with IMT, and a reverse correlation was observed between IMT with the percent of cardiac ejection fraction.

**
Conclusion:** Prevalence of thickening in intima-media complex is more evident in hemodialysis subjects with LVH. When there is LVH, IMT is similar in severity to the LVH.

**Implication for health policy/practice/research/medical education:**

Prevalence of thickening in intima-media complex is more evident in hemodialysis subjects with left ventricular hypertrophy. When there is left ventricular hypertrophy, intima-media complex thickening is similar in severity to the left ventricular hypertrophy.

## Introduction

Cardiovascular disease is the principal cause of morbidity and mortality in hemodialysis (HD) patients (1-2). The principal findings of cardiovascular disease are left ventricular hypertrophy (LVH), as determined by echocardiography (2,3), and arterial disease due to rapidly progressive atherosclerotic vascular disease (2-4), characterized by an enlargement and hypertrophy of arteries [intima-media complex thickening (IMT)] as can be determined by B-mode ultrasonography (2-4). LV mass increases progressively as renal function deteriorates and is exceedingly frequent in patients undergoing dialysis (1-3). Indeed, LVH and arterial disease are the two principal risk factors for cardiovascular mortality in hemodialysis patients (2-5). Carotid-IMT (c-IMT) is a marker of early atherosclerosis, its anatomic extent and progression, and IMT is increased in subjects with several risk factors and is a predictor of cardiovascular events and end-organ damage (6). Clinical manifestations of cardiovascular disease often arise in a stage of well-advanced atherosclerosis (6,7). However, arterial vessel wall changes occur during a presumably long sub-clinical lag phase characterized by functional disturbances and by gradual thickening of intima-media (6,7). IMT of large peripheral arteries, especially the carotid, can be assessed by B-mode ultrasound in a relatively simple way (6-8), thus the measurement of IMT has emerged as one of the methods of choice for determining early atherosclerotic changes, the anatomic extent of atherosclerosis and its progression and showing the effectiveness of medical therapy (6-8). Therefore, considerable attention has been directed toward cIMT by B-mode ultrasound, which can directly assess the IMT, corresponding to the thickness of the histologic intima and media (6-9). Bodies of evidence have shown that cIMT is a strong predictor of cardiovascular disease in the general population (6-8). However, the question remains as to whether ultrasonographic studies of cIMT are useful to find any relationship between with end-stage renal disease-related (ESRD-related) vascular changes as cIMT with LVH in HD patients (6-9). The cardiovascular mortality rate is elevated in those ESRD, diabetes mellitus and especially in those with diabetes mellitus and ESRD (1-4,6-9).

## Objectives

We studied a group of hemodialysis patients consisting of diabetics and non-diabetics to find an association between IMT with LVH and IMT with severity of hypertension and chest pain.

## Patients and Methods

### Patients

This cross-sectional study was done on 61 unselected patients ESRD, undergoing regular HD. Patient exclusion criteria were cigarette smoking, body mass index (BMI) more than 25, anti-lipid drug use, recent MI and vascular diseases as well as pericarditis and pericardial effusion in echocardiography. For stratification of hypertensive patients, according to the sixth and seventh report of the Joint National Committee on Prevention, Detection, Evaluation and Treatment of High Blood Pressure, we stratified hypertensive patients from stage one to three (stage zero equal to no HTN) (10). Stages of hypertension of HD patients were considered before treatment and at the start of hemodialysis treatment (10).

### Assessment of intima-media complex thickening

Carotid sonography was done by a single sonologist unaware of history or lab data of patients with a Honda-HS-2000 Sonograph and 7.5 MHz linear probe to measure IMT (4,8). The procedure was done at the end of diastolic phase; the sites of measurements were at the distal common carotid artery, area of bifurcation and at the proximal internal carotid artery (4,8). IMT was measured at the plaque-free areas with subjects in the supine position with neck hyperextension and head rotation for facilitation of the procedure. The carotids were evaluated in the longitudinal axis. By sonography, the carotid artery was found to have three different echoes: intima, as an echogenic layer line; media, as a hypoecho-layer; and an echogenic adventitia. IMT was defined as the distance from the leading edge of lumen-intima interface of the far wall to the leading edge of the media adventitia interface of the far wall. IMT more than 0.8 mm was considered abnormal (4,8) . A single cardiologist unaware of the patients’ data performed all the echo-cardiographies to determine left ventricular hypertrophy. On the basis of sepal thickness, we stratified the patients to no LVH (septal thickness between 6-11 mm), mild (septal thickness between 11-15 mm), moderate (septal thickness between 15-18 mm) and severe LVH (septal thickness>18 mm). LVH measurements were done at the end-diastolic phase (1-4,8). Cardiac ejection fraction between 55 to 75% was considered normal (1-4). We measured the mean right and left carotid IMT.

### Ethical issues

The research followed the tenets of the Declaration of Helsinki; written informed consent was obtained; and the research was approved by ethical committee of Shahrekord University of Medical Sciences.

### Statistical analysis

For statistical analysis, descriptive data are expressed as Mean±SD. Comparison between groups was performed using chi-squared test, Mann-Whitney U, as well as Kruskal-Wallis and Fisher’s exact test. For correlations, we used Spearman’s rho, Phi and Cramer’s V and also Eta tests. In addition, partial correlation test (with adjustment for age) was used. All statistical analysis was performed using SPSS version 11.00. Statistical significance was inferred at a p< 0.05.

## Results

The total number of patients was 61 (F=23, M=38), which consisted of 50 non-diabetic hemodialysis patients (F=20, M=30) and 11 diabetic hemodialysis patients (F=3, M=8). Table 1  shows the mean±SD of data; Tables 2 , 3  and 4  show the frequency distribution of chest pain, stages of HTN and stages of LVH in the patients. Mean±SD of the age of the subjects was 46.5±16 years. Mean±SD length of time, the patients had been on HD was 32±31 months. Mean±SD of cardiac ejection fraction was 51±8.9 percent. Also 39.3% of the patients had cardiac chest pain. In this study, there were no significant differences in age, percent of cardiac ejection fraction, cIMT and duration of HD between the males and females (p>0.05, Mann-Whitney U test). Also, there was no significant difference in LVH between the two sexes (chi-square test, p>0.05). There was no significant difference in the presence of chest pain or DM between the two sexes (Fisher’s exact test, p>0.05). No significant difference was found between gender and stages of hypertension (chi-square test, p>0.05). In this study, there was a positive correlation between stages of LVH and duration of HD treatment (p<0.01), However, no significant difference between stages of LVH and age (p>0.005, Kruskal-Wallis) was found. There was a positive relationship between stages of LVH and stages of HTN (r=0.580, p<0.001). There was no significant correlation between DM and LVH (Phi and Cramer’s V test, p>0.05). We found a significant difference between stages of LVH with cIMT (Kruskal-Wallis test, p>0.05). We found a significant positive correlation between presence of chest pain and DM (p<0.001), but no association between the presence of DM with presence of HTN, or gender with DM (Phi and Cramer’s V test, p>0.005). There was a significant difference of cIMT between the diabetic and non-diabetic groups (1.3±0.3 vs. 1±0.25 mm, respectively, p<0.05, Eta test). No correlation was found between DM and ejection fraction (p>0.005, Eta test), and no significant difference existed between duration of hemodialysis, age and ejection fraction between the diabetic and non-diabetic groups (p>0.05, Mann-Whitney U test). No significant correlation was found between ejection fraction and duration of hemodialysis treatment (p>0.05, Spearman’s rho). A significant correlation was observed between presence of chest pain and stages of LVH (p<0.001, Phi and Cramer’s V test). There was no difference between stages of HTN with presence of chest pain (p>0.05, chi-square test). Statistical analysis on cIMT with partial correlation test after adjustment for age showed no positive correlation between cIMT with duration of HD (p>0.05), and reverse correlation between cIMT with percent of ejection fraction (r = -0.353, p=0.005). Also, a positive association between cIMT and stages of HTN was observed (r =0.266, p=0.020).

**Table 1:** Mean±SD, minimum and maximum of data

		**Age** **years**	D.H.T* **months**	IMT** **mm**	EF*** **percent**dce6f2
Total Patients n=61	Mean±SD	46.5±16	32±31	1.06±0.3	51±8.9
	Min	15	2	0.50	25
	Max	78	108	1.70	70
Diabetic n=11	Mean±SD	57±16	22.6±22	1.3±0.3	
	Min	27	3	0.80	30
	Max	78	60	1.70	55
Non- diabetic N=50	Mean±SD	47.8±16	34±33	1±0.25	
	Min	15	2	0.50	25
	Max	78	108	1.60	70
*duration of HD, **carotid-IMT, *** ejection fraction					

**Table 2:** Frequency distribution of stages of HTN

**Stages of HTN**	**Total patients** **N=61**		DM group* N=11		**Non- DM group** **N=50**	
	No.	%	No.	%	No.	%
0	4	6.6	0	0	4	8
1	5	8.2	0	0	5	10
2	33	54.1	8	72.7	25	50
3	19	31.1	3	27.3	16	32

**Table 3:** Frequency distribution of chest pain in HD patients

**Chest pain**	**Total patients** **N=61**		**DM group** **N=11**	**Non- DM group** **N=50**
	No.	%	No.	%
Yes	24	39.3	9	81.8
No	34	60.7	2	18.2

**Table 4:** Frequency distribution of LVH in hemodialysis patients

	**Total patients** **N=61**		**DM group** **N=11**		**Non- DM group** **N=50**	
	No.	%	No.	%	No.	%
No LVH	9	14.8	1	9	8	16
Mild LVH	25	41	4	36.4	21	42
Mod LVH	20	32.8	4	36.4	16	36
Sever LVH	7	11.5	2	18.2	5	10

## Discussion

The principal findings of the present study were a positive correlation between stages of LVH with duration of HD, positive correlation between LVH with hypertension. Also, a positive correlation between stages of LVH with presence of chest pain, more thickening of the cIMT in the diabetic group was observed. We also found an association of diabetes mellitus with the presence of chest pain, a positive correlation between stages of HTN with cIMT. Finally, an inverse correlation between cIMT with percent of cardiac ejection fraction was observed too. Strauman *et al*. in a study on 62 patients on maintenance hemodialysis observed 65% prevalence of LV hypertrophy and showed that age, body mass index and duration of HTN were associated with LV hypertrophy and asymmetric septal hypertrophy (11). Greaves **et al*.* in the evaluation of 30 HD patients and 54 patients under peritoneal dialysis compared with 38 ESRD patients not yet on dialysis demonstrated that left ventricular wall thickness was greater in the dialysis group (12). Nishizawa *et al*. in a study on 438 patients with ESRD treated with hemodialysis showed a significantly greater risk for death from cardiovascular causes in patients who had significant higher IMT (13). Lin *et al*. in a research on forty normotensive HD patients demonstrated that LV mass was significantly positively related to cIMT (14). London *et al*. studied 70 uncomplicated ESRD patients and observed a significant correlation of ventricular wall thickness as well as left ventricular mass with cIMT (15). Zoccali *et al*. in an evaluation of 254 patients undergoing dialysis concluded that LV mass is a strong and independent predictor of survival and cardiovascular events in these patients (16). Muiesan *et al*. in a study to evaluate the structural association between the carotid artery and left ventricle in a general population in northern Italy showed that the common cIMT was significantly greater in subjects with concentric left ventricular hypertrophy (17). Our results provide the first direct evidence that diabetic patients with ESRD undergoing HD had more accelerated atherosclerosis and more involvement by ischemic heart disease (IHD) than non-diabetic hemodialysis patients. We found the association between cIMT and LVH and especially reverse correlation between cIMT with cardiac ejection fraction, meaning that thickening of the intima-media complex and cardiovascular involvement, especially LVH in hemodialysis patients, could have an accelerated atherosclerotic base, albeit other factors are involved. We have also demonstrated that cIMT is related to LVH, although the confirmation of this cardio-arterial interaction further highlights the importance of structural changes in large arteries in the pathogenesis of LVH in HD patients. Mallion believed that the prevalence of thickening in intima-media was more evident in subjects with LVH and that the presence of concentric remodeling of the left ventricle without LVH was associated with an increase in IMT (18).

## Conclusion

When there is LVH-in particular concentric- the IMT is similar in severity to the LVH. However, the question is whether carotid ultrasonography added relevant information to echocardiography-measured LVH in hemodialysis patients. Although cIMT has a prediction power for cardiovascular death independent of LVH, larger studies are needed to allow of better appreciation of the relative value of ultrasonography-measured IMT in these patients. Prevalence of thickening in intima-media complex is more evident in hemodialysis subjects with left ventricular hypertrophy. When there is left ventricular hypertrophy, intima-media complex thickening is similar in severity to the left ventricular hypertrophy.

## Authors’ contributions

HN conceptualized the study, obtained and analyzed the data, drafted the paper and gave final approval. MRK provided important intellectual input and helped in data acquisition and data analysis. MRK edited the draft.

## Conflict of interests

The author declared no competing interests.

## Ethical considerations

Ethical issues (including plagiarism, data fabrication, double publication) have been completely observed by the author.

## Funding/Support

None.
